# Binding Site Alteration Is Responsible for Field-Isolated Resistance to *Bacillus thuringiensis* Cry2A Insecticidal Proteins in Two *Helicoverpa* Species

**DOI:** 10.1371/journal.pone.0009975

**Published:** 2010-04-01

**Authors:** Silvia Caccia, Carmen Sara Hernández-Rodríguez, Rod J. Mahon, Sharon Downes, William James, Nadine Bautsoens, Jeroen Van Rie, Juan Ferré

**Affiliations:** 1 Department of Genetics, University of Valencia, Burjassot, Spain; 2 CSIRO Entomology, Canberra, Australia; 3 CSIRO Entomology, Narrabri, Australia; 4 Bayer BioScience, Gent, Belgium; Purdue University, United States of America

## Abstract

**Background:**

Evolution of resistance by target pests is the main threat to the long-term efficacy of crops expressing *Bacillus thuringiensis* (*Bt*) insecticidal proteins. Cry2 proteins play a pivotal role in current Bt spray formulations and transgenic crops and they complement Cry1A proteins because of their different mode of action. Their presence is critical in the control of those lepidopteran species, such as *Helicoverpa* spp., which are not highly susceptible to Cry1A proteins. In Australia, a transgenic variety of cotton expressing Cry1Ac and Cry2Ab (Bollgard II) comprises at least 80% of the total cotton area. Prior to the widespread adoption of Bollgard II, the frequency of alleles conferring resistance to Cry2Ab in field populations of *Helicoverpa armigera* and *Helicoverpa punctigera* was significantly higher than anticipated. Colonies established from survivors of F_2_ screens against Cry2Ab are highly resistant to this toxin, but susceptible to Cry1Ac.

**Methodology/Principal Findings:**

Bioassays performed with surface-treated artificial diet on neonates of *H. armigera* and *H. punctigera* showed that Cry2Ab resistant insects were cross-resistant to Cry2Ae while susceptible to Cry1Ab. Binding analyses with ^125^I-labeled Cry2Ab were performed with brush border membrane vesicles from midguts of Cry2Ab susceptible and resistant insects. The results of the binding analyses correlated with bioassay data and demonstrated that resistant insects exhibited greatly reduced binding of Cry2Ab toxin to midgut receptors, whereas no change in ^125^I-labeled-Cry1Ac binding was detected. As previously demonstrated for *H. armigera*, Cry2Ab binding sites in *H. punctigera* were shown to be shared by Cry2Ae, which explains why an alteration of the shared binding site would lead to cross-resistance between the two Cry2A toxins.

**Conclusion/Significance:**

This is the first time that a mechanism of resistance to the Cry2 class of insecticidal proteins has been reported. Because we found the same mechanism of resistance in multiple strains representing several field populations, we conclude that target site alteration is the most likely means that field populations evolve resistance to Cry2 proteins in *Helicoverpa* spp. Our work also confirms the presence in the insect midgut of specific binding sites for this class of proteins. Characterizing the Cry2 receptors and their mutations that enable resistance could lead to the development of molecular tools to monitor resistance in the field.

## Introduction

The agronomical impact of bioinsecticides based on *Bacillus thuringiensis* (*Bt*) has increased significantly since the late 1950s when commercial sprays based on this bacterium were first developed. Now *Bt* products are the most successful biopesticides used in agriculture, forestry and public health [1]. However, the major current interest of *Bt* insecticidal proteins (Cry proteins) resides on the large scale use of insect-resistant engineered plants expressing these proteins. Such genetically modified *Bt-*crops today represent the most widely adopted transgenic crops after those with herbicide tolerance [2].

*Bt*-cotton expressing Cry1Ac was first adopted in 1996 in the US (as Bollgard) and Australia (as Ingard). This *Bt*-cotton was developed to target key pests highly susceptible to Cry1Ac, such as the tobacco budworm, *Heliothis virescens*, and pink bollworm, *Pectinophora gossypiella*, and also to control the less susceptible cotton bollworms of the *Helicoverpa* genus (mainly *H. zea* and *H. armigera*). To improve the effectiveness and to delay resistance evolution by targeted Lepidoptera, a second generation *Bt*-cotton (Bollgard II) was developed that expressed Cry1Ac and Cry2Ab toxins. Bollgard II was approved for commercial use in 2002 and introduced the following year in the US and Australia. Within one year of the introduction of this variety in Australia, Ingard was removed from sale to preserve the susceptibility of the major pests *H. armigera* and *H. punctigera* to Cry1Ac [2]. US growers planted cotton producing only Cry1Ac along with Bollgard II for seven years (from 2003 to 2009), which may have resulted in an increase in the LC50's of populations of *H. zea* over time that has been ascribed by Tabashnik et al. 2009 [3] to be due to resistance.

The pyramiding strategy combining different *cry* genes serves several purposes: to broaden the insecticidal spectrum, to increase the effectiveness of the plant for the less susceptible insect species, and to delay the development of resistance [4]–[6]. Early studies on the mode of action of Cry1A and Cry2A proteins [7], along with the general lack of cross-resistance [reviewed in 5], [6], suggested that they had different targets in the insect midgut. Recently, independent high affinity binding sites for Cry1Ac and Cry2Ab were demonstrated in *H. armigera* and *H. zea* [8], [9]. Consequently, plant varieties expressing these two toxins should be far more effective in delaying or even avoiding the evolution of resistance than a single toxin product [4].

Understanding the mechanism of resistance to Cry2A proteins is of special interest in the light of the recent reported cases of field-evolved resistance to Bt-crops expressing Cry1 proteins. Cry1Ab corn in South Africa failed to control *Busseola fusca* [10] and the same occurred for Cry1F corn against *Spodoptera frugiperda* in Puerto Rico [11]. Although field failures of Bollgard II plants have not been reported, results from laboratory diet bioassays estimating LC_50_ values suggest that susceptibility of *H. zea* to Cry1Ac and Cry2Ab has decreased in the southeastern US [3], [12]–[15].

Regardless of previous exposure to Cry1Ac through spray formulations and Ingard cotton, resistance monitoring data in Australian cotton fields suggest that in the major targets *H. armigera* and *H. punctigera* alleles which confer resistance to Cry1Ac were initially rare [16], [17]. This conclusion is based on a large number of screens performed from 2002 to 2006 of both *Helicoverpa* spp. that detected no Cry1Ac resistance alleles (estimated frequency  = 0), resulting in upper limits of the estimated frequencies of 0.0003 for *H. armigera* (n = 3304 alleles) and 0.0005 for *H. punctigera* (n = 2180 alleles). Surprisingly, prior to the widespread adoption of Bollgard II in 2004/2005, a relatively high frequency of recessive alleles for Cry2Ab resistance was found in both *Helicoverpa* species using F_2_ screens (0.0018–0.0033) [16], [17].

Laboratory selected insects have provided evidence for several mechanisms of resistance to Cry proteins, though altered binding to midgut receptors is the most common one to confer high levels of resistance to Cry1A proteins [5], [6]. It is noteworthy that, without exception, this mechanism of resistance to Cry1A proteins has been the major one found in insect populations that have developed resistance to *Bt* commercial formulations under field or semi-field (greenhouse) conditions [18]–[25]. So far, the mechanism of resistance has not been studied yet in those cases of field-evolved resistance to *Bt* crops. Despite the key role that Cry2Ab is currently playing and is likely to play in future insect-resistant crops, few studies have dealt with resistance to Cry2 proteins and nothing is known about the possible underlying biochemical and/or physiological mechanisms of resistance to this class [5], [6].

In this article we present results on the characterization of the field-isolated resistance to Cry2Ab in *H. armigera* and *H. punctigera.* We previously demonstrated for both species that these Cry2Ab resistant insects are susceptible to Cry1Ac [26], [27], and for *H. armigera* that Cry2Ab resistant insects are cross-resistant to Cry2Aa [26]. Herein we report results from additional bioassays with Cry1Ab and Cry2Ae, as well as analyses to determine whether Cry2Ab binding was reduced in the field-isolated resistant insects and if the occurrence of shared binding sites could account for the cross-resistance patterns that we observed.

## Results

### Susceptibility assays

Surface treatment bioassays performed on neonates of Cry2Ab resistant *H. armigera* and *H. punctigera* showed that these strains were completely resistant to the maximum concentration of Cry2Ab employed (Table 1). Mortality at the maximum concentration was low for the *H. armigera* resistant strain SP15 (overall, 5%) and for the *H. punctigera* resistant strain Hp4-13 (6%), and in both species was similar to that in the control treatment. Furthermore, the Cry2Ab resistant strains of both species were similarly resistant to Cry2Ae (Table 1). It had been demonstrated previously that SP15 was cross-resistant to Cry2Aa [26]. Insects from all strains tested were susceptible to Cry1Ab and Cry1Ac (Table 1) [27], [28]. In previous work the *H. armigera* strain 6–364 strain was found to be allelic to SP15 in complementation tests [29] and when isolated from F_2_ screens was fully susceptible to Cry1Ac.

**Table 1:** Bioassays with Cry2Ab resistant and susceptible *H. armigera* and *H. punctigera.*

Species	Strain	Toxin	LC_50_ a(95% CI)	Slope ± SE
*H. armigera*	GR susceptible	Cry1Ab	0.17 (0.09–0.34)	1.14 ± 0.13
		Cry1Ac^b^	0.011 (0.01–0.02)	1.23 ± 0.12
		Cry2Ab	0.13 (0.09–0.18)	1.81 ± 0.22
		Cry2Ae	0.13 (0.10–0.18)	1.80 ± 0.20
	ANGR susceptible	Cry1Ab	0.13 (0.11–0.16)	1.82 ± 0.17
		Cry1Ac^c^	0.07 (0.05–0.09)	1.7 ± 0.1
		Cry2Ab	0.06 (0.04–0.07)	2.6 ± 0.27
		Cry2Ae	0.06 (0.05–0.08)	2.02 ± 0.19
	SP15 resistant	Cry1Ab	0.09 (0.06–0.12)	2.07 ± 0.16
		Cry1Ac^b^	0.02 (0.01–0.02)	1.23 ± 0.12
		Cry2Ab	>250^d^	-
		Cry2Ae	>250^d^	-
*H. punctigera*	LHP susceptible	Cry1Ab	0.73 (0.46–1.42)	1.10 ± 0.14
		Cry1Ac^e^	0.12 (0.08–0.21)	1.00 ± 0.11
		Cry2Ab	0.06 (0.04–0.10)	1.42 ± 0.13
		Cry2Ae	0.08 (0.05–0.10)	1.77 ± 0.19
	Hp4.13 resistant	Cry1Ab	0.24 (0.14–0.37)	1.75 ± 0.17
		Cry1Ac^e^	0.19 (0.15–0.26)	2.01 ± 0.25
		Cry2Ab	>250^d^	-
		Cry2Ae	>250^d^	-

a Values are in µg/cm^2^.

b Data from [26].

c Data from [28].

d LC_50_ and slopes could not be calculated as there was little or no mortality at the maximum concentration tested (0.25 mg/cm^2^).

e Data from [27].

### Binding of ^125^I-Cry proteins to brush border membrane vesicles (BBMV) from *H. armigera*

To determine whether reduced binding of Cry2A proteins could be the underlying mechanism of Cry2Ab resistance in the *H. armigera* strains, Cry2Ab and Cry1Ac (as a control) were labeled with ^125^I and specific binding to BBMV from susceptible (GR and ANGR) and resistant (SP15 and 6–364) *H. armigera* strains was tested.

**Figure 1:**
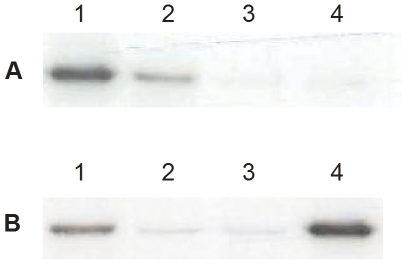
Binding of ^125^I-Cry2Ab proteins to BBMV from *Helicoverpa spp.* revealed by autoradiography. ^125^I-Cry2Ab was incubated with BBMV in the absence or the presence of an excess of competitor, and the pellet obtained after centrifuging the reaction mixture was subjected to SDS-PAGE and exposed to an X-ray film for 10 days. (A) ^125^I-Cry2Ab binding to *H. armigera*: lane 1, a sample of ^125^I-Cry2Ab protein used in the binding assays; lane 2, ^125^I-Cry2Ab incubated with BBMV in the absence of competitor; lane 3, homologous competition (excess of unlabeled Cry2Ab); lane 4, ^125^I-Cry2Ab incubated with BBMV from SP15-resistant insects. (B) ^125^I-Cry2Ab binding to *H. punctigera*: lane 1, ^125^I-Cry2Ab incubated with BBMV in the absence of competitor; lane 2, homologous competition; lane 3, ^125^I-Cry2Ab incubated with BBMV from Hp4-13-resistant insects; lane 4, a sample of the ^125^I-Cry2Ab protein used in the binding assays.

As a first approach, binding of Cry2Ab was tested by incubating BBMV from the GR strain with radiolabeled Cry2Ab (Fig. 1A). An expected band of 49 kDa was observed [9], corresponding to the binding of ^125^I-Cry2Ab to BBMV from susceptible insects (Fig. 1A, lane 2). Co-incubation with an excess of unlabeled Cry2Ab reduced binding of ^125^I-Cry2Ab (Fig. 1A, lane 3), indicating that most of this binding was specific. However for BBMV from the resistant SP15 strain, ^125^I-Cry2Ab failed to bind (Fig. 1A, lane 4). This result demonstrates that specific binding sites for Cry2Ab are altered in resistant insects.

**Figure 2:**
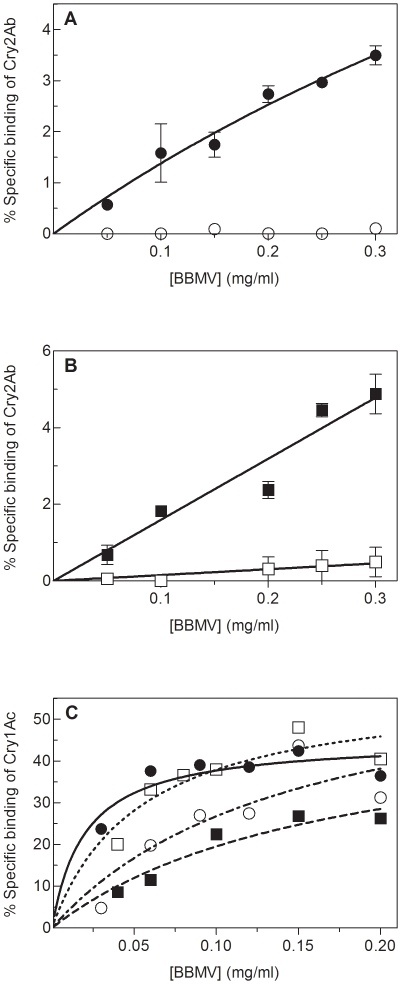
Binding of ^125^I-Cry proteins to BBMV from *H. armigera*. Binding of iodinated Cry proteins to *H. armigera* at increasing concentrations of BBMV from GR (•) and ANGR (▪) susceptible strains, and to SP15 (○) and 6-364 (□) resistant strains. Non-specific binding was determined by adding an excess of unlabeled protein to the reaction. Specific binding was calculated by subtracting the non-specific binding from the total binding. (A) Specific binding of ^125^I-Cry2Ab to BBMV from SP15 and its susceptible control (GR) strain. (B) Specific binding of ^125^I-Cry2Ab to BBMV from 6-364 and its susceptible control (ANGR) strain. (C) Specific binding of ^125^I-Cry1Ac. Data points in figures A and B represent the means of two replicates.

In a second approach, a fixed concentration of labeled protein was incubated with increasing concentrations of BBMV from each strain (Fig. 2). Non-specific binding was determined by adding an excess of unlabeled protein and specific binding was calculated by subtracting the non-specific binding from the total binding. In the two susceptible strains, an increase in the specific binding of ^125^I-Cry2Ab was observed corresponding to the increase of BBMV concentration (Fig. 2A and 2B). In contrast, in the two resistant strains, specific binding of Cry2Ab was either totally absent or highly reduced (Fig. 2A and 2B). In the case of ^125^I-Cry1Ac, the specific binding was not substantially different for the susceptible and resistant strains at increasing concentrations of BBMV (Fig. 2C). These results indicate that resistance to Cry2Ab is due to the lack of specific high affinity binding sites for Cry2Ab in the midgut. Binding of Cry1Ac in the resistant insects remains unaltered, confirming that Cry1Ac binding sites are not shared with those of Cry2Ab. This is in agreement with the lack of cross-resistance between these two proteins (Table 1) and the binding site model for this species [8], [9].

### Binding of ^125^I-Cry proteins to BBMV from *H. punctigera* strains

**Figure 3:**
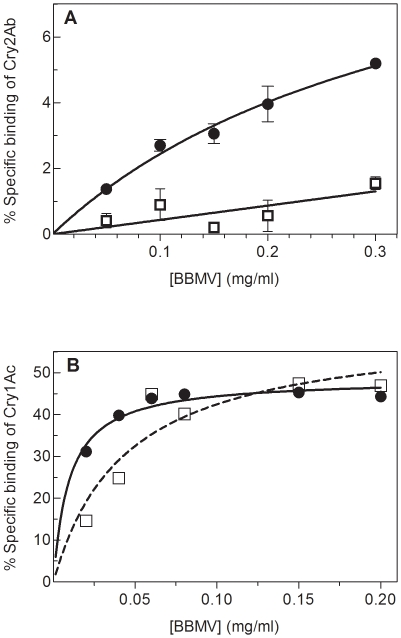
Binding of ^125^I-Cry proteins to BBMV from *H. punctigera*. Binding of iodinated Cry proteins to *H. punctigera* at increasing concentrations of BBMV from the susceptible LHP strain (•) and the resistant Hp4-13 strain (□). Non-specific binding was determined by adding an excess of unlabeled protein to the reaction. Specific binding was calculated by subtracting the non-specific binding from the total binding. (A) Specific binding of ^125^I-Cry2Ab. (B) Specific binding of ^125^I-Cry1Ac. Data points in figure A represent the means of two replicates.

Similar binding experiments as described for *H. armigera* above were carried out with a susceptible (LHP) and a Cry2Ab resistant (Hp4-13) strain of *H. punctigera*. Qualitative experiments with ^125^I-Cry2Ab showed binding of this protein to BBMV from susceptible larvae (Fig. 1B, lane 1) that was displaced by unlabeled Cry2Ab (Fig. 1B, lane 2). Absence of ^125^I-Cry2Ab binding was observed in resistant insects (Fig. 1B, lane 3). When binding of ^125^I-Cry2Ab was measured at increasing concentrations of BBMV, specific binding increased in the susceptible strain in a dose dependent manner, however, a very strong reduction in binding was observed with BBMV from the resistant strain (Fig. 3A). No marked differences in binding were found for ^125^I-Cry1Ac between resistant and susceptible strains (Fig. 3B). These results, like those for *H. armigera,* show that an alteration in Cry2Ab binding is responsible for the resistance to this protein and that Cry2Ab binding sites are different from Cry1Ac sites, which remain unaltered in the resistant insects. The pattern of binding or lack of binding is in agreement with the susceptibility pattern shown in Table 1.

### Competition experiments with *H. punctigera* BBMV

To unravel the binding site specificity of the Cry proteins under study in *H. punctigera*, binding of ^125^I-Cry2Ab and ^125^I-Cry1Ac was measured in the presence of unlabeled Cry proteins as competitors.

**Figure 4:**
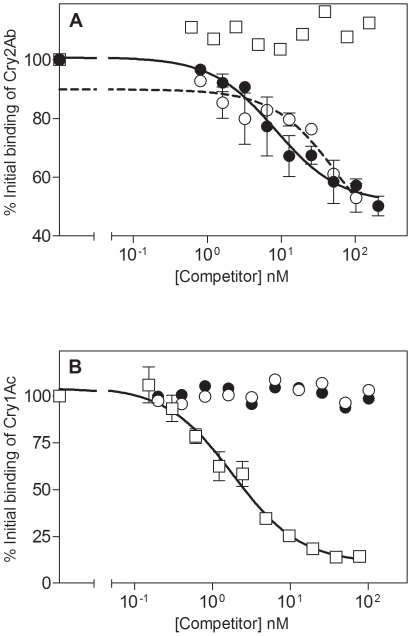
Competition binding experiments with *H. punctigera* BBMV. Binding of ^125^I-Cry2Ab (A) and ^125^I-Cry1Ac (B) to BBMVs from *H. punctigera* at increasing concentrations of unlabeled competitor: Cry2Ab (•), Cry2Ae (○), and Cry1Ac (□).

Homologous competition assays using ^125^I-Cry2Ab (and unlabeled Cry2Ab) confirmed that this protein binds saturably to *H. punctigera* BBMV (Fig. 4A). Binding parameters determined from this experiment showed that binding was of high affinity (*K*_d_ = 6.5±1.6 nM) with an *R*_t_ value of 2.1±0.4 pmol per mg of BBMV (Table 2). Competition binding assays using Cry2Ae as a heterologous competitor showed that this protein readily competed with ^125^I-Cry2Ab (Fig. 4A). In contrast, unlabeled Cry1Ac was unable to compete for ^125^I-Cry2Ab binding in the range of concentrations tested. These results indicate that Cry2Ab binding sites are shared with Cry2Ae, but not with Cry1Ac.

**Table 2:** Binding parameters in *H. punctigera* a.

Toxin	*K*_d_ (nM)	*R*_t_ (pmol/mg) b
Cry1Ac	0.75 ± 0.10	11.6 ± 1.0
Cry2Ab	6.5 ± 1.6	2.1 ± 0.4

a Mean ± SEM.

b Values are expressed in picomoles per milligram of BBMV protein.

Competition assays were also carried out with ^125^I-Cry1Ac using Cry1Ac, Cry2Ab and Cry2Ae as competitors (Fig. 4B). Binding parameters obtained from homologous competition experiments are given in Table 2. Cry2A proteins did not compete for binding with ^125^I-Cry1Ac, confirming the occurrence of different binding sites for Cry1Ac and Cry2A proteins in this insect species, and in agreement with the susceptibility pattern observed in the resistant insects.

## Discussion

Since reduced binding is a major mechanism of resistance to Cry1A proteins [5], [6], and because the occurrence of Cry2A specific binding sites has been recently established in *H. armigera* and *H. zea* [8], [9], we wanted to assess whether altered Cry2Ab binding would explain the field-isolated Cry2Ab resistance. BBMV prepared from *H. armigera* and *H. punctigera* resistant insect larvae had essentially lost the capacity to bind Cry2Ab, but could readily bind Cry1Ac. These results indicate that an alteration in the Cry2A receptor/s is responsible for conferring resistance to these proteins and that non-specific or non-saturable binding of Cry2A to the insect midgut is not involved in toxicity of Cry2A proteins as earlier proposed [7]. Since Cry2Ab and Cry2Ae, but not Cry1Ac, share binding sites in both *Helicoverpa* species, the alteration of the receptor for Cry2A proteins is expected to confer resistance simultaneously to these two proteins without affecting the insect susceptibility to Cry1A proteins. This is in agreement with the bioassay data in Table 1. It should be mentioned that, although a large percentage of Cry2Ab binding in experiments presented herein seems to be non-specific, it is very likely that most of the radioactivity catalogued as non-specific binding is actually radioactivity coming from precipitated labeled-Cry2Ab [9].

Our results with field isolated resistance differ from other studies which dealt with cross-resistance between Cry1A and Cry2A proteins in laboratory selected strains. Laboratory selection of a *H. virescens* strain with Cry1Ac conferred moderate levels of resistance to Cry2Aa and to a number of Cry1 proteins, and the inheritance of resistance appeared to be polygenic [30], [31]. In a study performed on field collected *H. armigera* in China low levels of tolerance to Cry2Ab were found in insects collected from Cry1Ac-cotton fields; tolerance to Cry1Ac and Cry2Ab was positively correlated and it was suggested to represent the cumulative effect of multiple minor resistance genes [32]. Just as low-level broad-spectrum resistance is typical of the additive effects of multiple loci, high-level narrow-spectrum resistance suggests the involvement of major genes, as seems to be the case in the field derived resistant populations studied herein. Recently, resistance to Cry1Ac and Cry2Ab was obtained upon laboratory selection with Cry2Ab of a *Pectinophora gossypiella* population already carrying Cry1Ac resistance alleles; based on the high levels of resistance and previous data on the mode of action of the two proteins, the dual resistance was probably due to the combined action of resistance alleles at two independent loci [33], [34].

A key step in the mode of action of *Bt* insecticidal Cry proteins is the binding to specific sites in the brush border membrane of the larval midgut [35], [36]. Significantly reduced binding of insecticidal proteins from the Cry1A family has been found in several insect species selected for resistance to *Bt*: *Plodia interpunctella* [37], [38], *Plutella xylostella* [18]–[23], [25], *H. virescens* [31], [39], *P. gossypiella* [33], *H. armigera* [40], and *Trichoplusia ni* [24]. This type of altered target site mechanism has not been previously shown for other Cry proteins, although it has indirectly been proposed for Cry1F and Cry1J in *P. xylostella* [23], [41] and *H. virescens* [42]. In some, but not in all cases, the lack of binding has been shown to be linked with mutations in a cadherin gene [43]–[46].

The recent demonstration that Cry2A toxins bind to specific sites located in the brush border of the midgut of *H. armigera* [8], [9] and *H. zea* [9] also showed that these sites were different from those of Cry1A proteins. Herein we extended these data with a new species, *H. punctigera*, and show that, like *H. armigera*: (i) Cry2Ab binds saturably and with high affinity to sites in the brush border membrane, (ii) Cry2Ae competes for the same binding sites, and (iii) these sites are not recognized by Cry1Ac, which has independent high affinity binding sites. This pattern of binding sites predicts that resistance to one class of Cry proteins (i.e., Cry1A) can occur without affecting the other class (i.e., Cry2A) and vice versa. These results, along with the demonstration that binding can be lost to one class of toxins (e.g., Cry2A) without affecting binding to the other class (e.g., Cry1A) strongly supports the strategy of pyramiding *cry1A* and *cry2A* genes in transgenic plants.

Our results show that binding site alteration, as a mechanism of resistance, is not restricted to the Cry1A class of proteins, but can also extend to the Cry2A class. Because we examined multiple Cry2A-resistant strains carrying resistance alleles present in field populations, we conclude that binding site alteration is the most likely means that field populations evolve resistance to Cry2 proteins in *Helicoverpa* spp. Based on the present and previous studies, it is likely that changes in midgut binding sites will prove to be the most common means that insects evolve field resistance to *Bt* insecticidal proteins.

The confirmation of the presence in the insect midgut of specific binding sites for the Cry2A class of proteins leads to the interest to characterize the receptors in the light of developing molecular tools for monitoring the evolution of resistance in the field (e.g., [47], [48]).

## Materials and Methods

### Insect strains

The Cry2Ab resistant strains SP15, 6–364 (*H. armigera*) and Hp4-13 (*H. punctigera*) were isolated from Australian field populations using an F_2_ screen in 2002, 2006 and 2004, respectively. Each resistant strain was established from a single pair of moths. Progeny from the pair were allowed to mate together and the colony was formed from F_2_ offspring that survived a discriminating concentration of Cry2Ab. Details of the proceedures and toxin used to establish resistant *H. armigera* and *H. punctigera* strains and the origin of susceptible strains are presented in Mahon et al. [16] and Downes et al. [17].

The resistant strains examined are typical of similar field isolates of both species in which resistance is due to alleles at the same locus [29, Downes et al., unpublished]. Resistance is recessive and insects from resistant strains are extremely tolerant to high levels of Cry2Ab toxin [26], [27].

The GR and ANGR strains (*H. armigera*) and LHP (*H. punctigera*) are susceptible to Cry1Ac and Cry2Ab toxins [26], [27, see also below]. This susceptibility is monitored regularly, including prior to the experiments reported herein, by evaluating responses to discriminating doses of these toxins that kill ∼95% of susceptible neonate larvae.

Since the resistant strains established from the F_2_ screen initially possessed a very restricted gene pool they were crossed to the susceptible strains, maintained without selection for one generation, and re-selected with Cry2Ab. The SP15, 6–364, and Hp4-13 resistant strains had been outcrossed seven (to GR), four (to ANGR), and five (to LHP) times, respectively. This method maintained fitness in the resistant strains and produced a colony that was presumed to be near isogenic with the corresponding susceptible strain.

### Bioassays

Surface contamination bioassays were performed with Cry2Ab resistant and susceptible neonates using methods outlined in Mahon et al. [26]. At least three replicate assays were performed for each toxin and strain evaluated. Each assay assessed the response of 22–45 insects to the toxin at each concentration. For each assay, diet presented to a similar number of neonates remained untreated to assess control mortality. Analyses were conducted using Polo Plus [49]. Data presented represent the combined data from replicates.

#### *B. thuringiensis* Cry proteins

The Cry1Ab, Cry1Ac, Cry2Ab, and Cry2Ae were obtained from recombinant *E. coli* strain WK6 harbouring plasmid pMAAB expressing Cry1Ab [50], *B. thuringiensis* strain HD73 (*Bacillus* Genetic Stock Collection, Columbus, OH), recombinant *B. thuringiensis* strain BtIPS78/11 [51] and recombinant *B. thuringiensis* subsp. *berliner* 1715 Cry^−^ mutant (Institut Pasteur, Paris) harbouring plasmid pGA32 expressing Cry2Ae, respectively, as previously described [9].

### Midgut isolation and BBMVs preparation

Last instar larvae of *H. armigera* and *H. punctigera* were dissected and the midguts lyophilized. BBMV were prepared from lyophilized midguts [52] by the differential magnesium precipitation method [53], frozen in liquid nitrogen and stored at −80°C. The protein concentration of the BBMV preparations was determined by the method of Bradford [54] using bovine serum albumin as a standard.

### Radiolabeling of Cry proteins

Cry1Ac and Cry2Ab proteins were labeled using the chloramine T method as previously described [9], [55]. The purity of the labeled proteins were checked by analyzing the elution fractions by SDS-PAGE with further exposure at −20°C of the dry gel to an X-ray film.

### Binding assays with ^125^I-labeled Cry1Ac and Cry2Ab

Prior to being used, BBMV were centrifuged for 10 min at 16000 × *g* and resuspended in binding buffer (8 mM Na_2_HPO_4_, 2 mM KH_2_PO_4_, 150 mM NaCl; pH 7.4; 0.1% bovine serum albumin).

To check the presence of specific binding and determine the optimal concentration of BBMV to use in competition experiments, increasing amounts of BBMV were incubated with either 0.28 nM or 0.04 nM of labeled Cry1Ac and Cry2Ab, respectively, in a final volume of 0.1 ml of binding buffer for 1 h at 25°C. An excess of unlabeled toxin (0.4 µM) was used to calculate the non-specific binding. After incubation, samples were centrifuged at 16000 × *g* for 10 min and the pellet was washed with 500 µl of cold binding buffer. The radioactivity retained in the pellet was measured in an LKB 1282 Compugamma CS gamma counter. Specific binding was calculated by subtracting the non-specific binding from the total binding.

Competition experiments were done by incubating either 20 µg of BBMV and 0.28 nM ^125^I-Cry2Ab, or 5 µg of BBMV and 0.04 nM ^125^I-Cry1Ac, in a final volume of 0.1 ml of binding buffer for 1 h at 25°C in the presence of increasing amounts of unlabeled Cry proteins. The reaction was stopped by centrifugation as described above. For quantitative assays, the fraction of labeled protein bound to BBMV was determined in a gamma counter. Dissociation constants and concentration of binding sites were calculated using the LIGAND program [56]. For qualitative assays, the pellets were boiled for 10 min in loading buffer and run in SDS-PAGE. The labeled protein retained in the pellet was detected by autoradiography after 10 days of exposure at −20°C.
